# Comparison of the linkage results of two phenotypic constructs from longitudinal data in the Framingham Heart Study: analyses on data measured at three time points and on the average of three measurements

**DOI:** 10.1186/1471-2156-4-S1-S20

**Published:** 2003-12-31

**Authors:** Rong Cheng, Naeun Park, Susan E Hodge, Suh-Hang Hank Juo

**Affiliations:** 1 Columbia Genome Center, Columbia University, 1150 St. Nicholas Avenue, New York, New York, USA; 2 New York State Psychiatric Institute, Columbia University, 1051 Riverside Drive, New York, New York, USA; 3 Department of Epidemiology, Columbia University, New York, New York, USA

## Abstract

**Background:**

Family studies are often conducted in a cross-sectional manner without long-term follow-up data. The relative contribution of a gene to a specific trait could change over the lifetime. The Framingham Heart Study offers a unique opportunity to investigate potential gene × time interaction. We performed linkage analysis on the body mass index (BMI) measured in 1970, 1978, and 1986 for this project.

**Results:**

We analyzed the data in two different ways: three genome-wide linkage analyses on each exam, and one genome-wide linkage analysis on the mean of the three measurements. Variance-component linkage analyses were performed by the SOLAR program. Genome-wide scans show consistent evidence of linkage of quantitative trait loci (QTLs) on chromosomes 3, 6, 9, and 16 in three measurements with a maximum multipoint LOD score > 2.2. However, only chromosome 9 has a LOD score = 2.14 when the mean values were analyzed. More interestingly, we found potential gene × environment interactions: increasing LOD scores with age on chromosomes 3, 9, and 16 and decreasing LOD scores on chromosome 6 in the three exams.

**Conclusion:**

The results indicate two points: 1) it is possible that a gene (or genes) influencing BMI is (are) up- or down-regulated as people aged due to aging process or changes in lifestyle, environments, or genetic epistasis; 2) using mean values from longitudinal data may reduce the power to detect linkage and may have no power to detect gene × time, and/or gene × gene interactions.

## Background

An advantage of the Framingham Heart Study is its repeated measurement of several cardiovascular risk factors over a long period of time. The Framingham Heart Study offers a unique opportunity to investigate the value of follow-up family studies. Quantitative trait data from repeated measurements in follow-up studies often fluctuate due to changes in lifestyle, age-related covariates, gene × gene interaction, and measurement errors. Although there are statistical tests, such as the generalized estimating equations (GEE) model [1] to analyze longitudinal data, there are no similar methods to analyze longitudinal family data. Thus, several linkage studies using the Framingham Heart Study performed analyses on the mean values [2]. We wondered whether it would be advantageous to perform separate linkage analysis for each measurement at different time points, rather than just use the mean values. Body mass index (BMI) is a good example of a quantitative trait that has high heritability, fluctuates due to biological reasons, but has minimal measurement errors. In this study, we compared the results from two analyses. In the first method we performed linkage analyses on the mean BMI from three measurements (in 1970, 1978, and 1986); then we conducted separate linkage analyses on each measurement. Advantages and disadvantages will be discussed by comparing these two approaches.

## Methods

In this study 330 pedigrees from the Framingham Heart Study from Genetic Analysis Workshop 13 data were used. While most pedigrees consist of 4 to 10 subjects in two generations, there are also several large pedigrees (up to 29 participants) and a few pedigrees that include three generations. The pedigrees consist of 4692 subjects, of whom 2885 have phenotype data. Cleaned genotyping data with 401 molecular markers are provided for chromosomes 1 through 22 for 1702 of the 4692 subjects.

We restricted our focus to BMI (kg/m^2^). All data including BMI, high-density lipoprotein-cholesterol (HDL-C, mg/dl), age, sex, cigarette smoking, and alcohol consumption were first explored to see their distributions and outliers. The BMI data were available from years 1970, 1978, (1976 for the Framingham Heart Study Cohort 1 and 1978 for Cohort 2), and 1986. If the data were missing in year 1970, the information was supplied from the data in 1968. The mean BMI was calculated from three time points (years 1970, 1978, and 1986). We took the mean of three exams for each individual (if a person had only two exams, we took the average of those two). Covariates, age, sex, smoking, alcohol, HDL-C, and interactions between HDL-C and age, smoking, and sex were included in the linkage analyses. Mean HDL-C and mean age were calculated in same way. Due to many nonsmokers and non-alcohol drinkers, the smoking and alcohol data were highly skewed. Therefore, these two variables were recoded as categorical variables. Smoking information was equally divided into four categories, and alcohol consumption into five categories. Four different genome-wide linkage analyses were performed for the three individual measurement and the mean values. The program SOLAR version 1.7.4 [3] was used for heritability estimation. In the SOLAR program, heritability is estimated by the multiple regression method. This program uses the general pedigree variance-component (VC) analysis [4] and extended multipoint identity-by-descent (IBD) estimation methods for quantitative trait locus (QTL) mapping.

## Results

There were a few outliers (> 3 SD: 24, 25, 24, and 27 outliers for 1970, 1978, 1986 and the mean, respectively, and most of them were between 3 SD and 4 SD) in BMI. First we performed the genome scan with outliers, and then analyses without outliers were carried out for four candidate chromosomal regions. Table 1 shows the mean ± SD, minimum, maximum, sample size, and estimated heritability ± standard error (SE) in the three time points and mean values of the three exams. When the analyses were performed with outliers, the estimated heritabilities of BMI ranged from 40.55 to 44.80. Heritability estimate with outliers from mean values of BMI was higher and had smaller SE than any three heritabilities estimated from each measurement.

**Figure 1:**
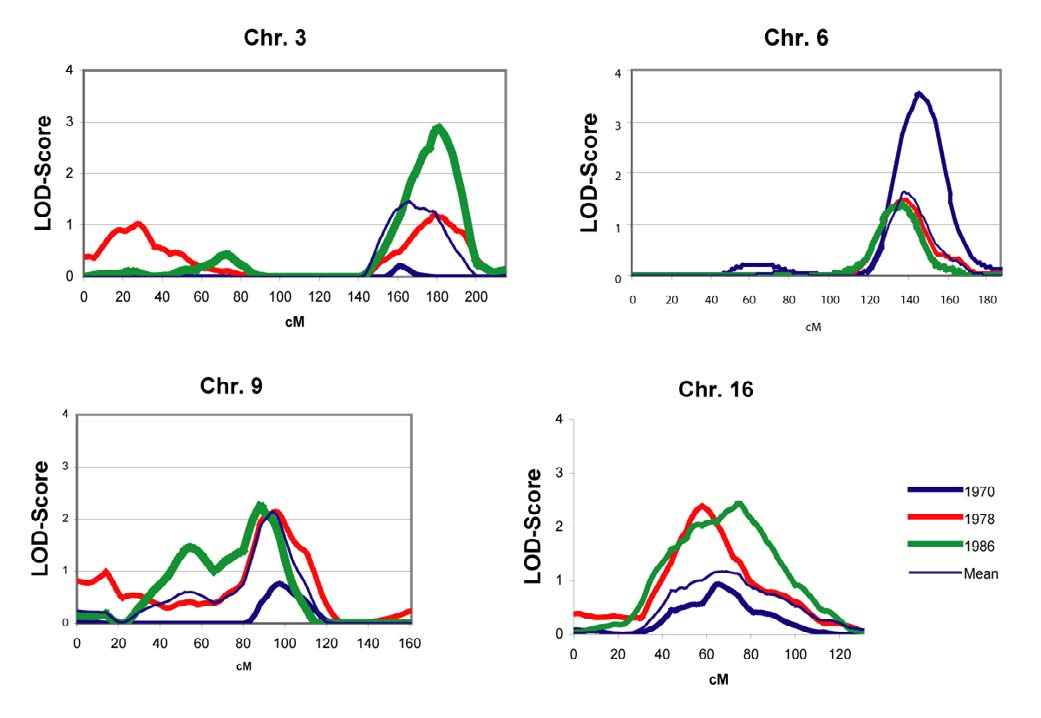
Multipoint variance-component linkage analysis results with outliers for time points 1970, 1978, 1986, and mean BMI of the three exams. LOD scores on chromosomes 3, 6, 9, and 16 were calculated by the SOLAR version 1.7.4 software package.

We considered "evidence" of linkage if a LOD score was ≥ 2.2 in one or more of the four analyses (three individual measurements plus mean values). The results of the genome scan with outliers showed that chromosomes 3, 6, 9, and 16 have evidence of quantitative trait loci (QTLs) affecting BMI (Fig. 1). The peaks with the highest LOD scores when outliers were included in these four regions were located at 181 cM of chromosome 3 (LOD 2.88 in 1986), 146 cM of chromosome 6 (LOD 3.53 in 1970), 88 cM of chromosome 9 (LOD 2.24 in 1986), and 75 cM of chromosome 16 (LOD 2.43 in 1986), respectively. When outliers were excluded, peaks with the highest LOD scores were located at 181 cM chromosome 3 (LOD 0.93 in 1986), 138 cM of chromosome 6 (LOD 0.92 in 1978), 97 cM of chromosome 9 (LOD 4.32 in 1978), and 58 cM of chromosome 16 (LOD 2.22 in 1978), respectively. In Table 2, the chromosomal locations and LOD scores at the highest peaks are summarized. Wu et al. [5] recently performed a combined analysis of genome scans and meta-analysis on 6849 individuals from four ethnic groups (White, Black, Mexican-American, and Asian), and this was the largest combined data set examined thus far. Our findings on chromosomes 3 (~180 cM) and 16 (~75 cM) replicate two of their findings. In addition, Duggirala et al. [6] reported that obesity is linked to chromosome 6 (~150 cM) in Mexican-Americans. Atwood et al. [7] more recently analyzed BMI data of six time points from the Framingham Heart Study, and found linkage evidence in same chromosomal regions. Lindgren et al. [8] reported linkage of type 2 diabetes on chromosome 9 (between 56–76 cM) in Finnish families. However, only chromosome 9 yielded a LOD score = 2.14 when mean values of BMI with outliers were used for analysis. This indicates potential type II errors when only mean values are analyzed.

The most interesting finding is an apparent pattern of either increasing or decreasing LOD scores when outliers were included, in the three exams on these four chromosomes 3, 6, 9, and 16 (Fig. 1). In the region of 155~190 cM on chromosome 3, the LOD scores obviously increased with age. A similar trend occurred in the regions of 80~110 cM on chromosome 9, and 50~80 cM on chromosome 16, but the increasing scale between 1978 and 1986 was smaller than that on chromosome 3. In contrast, in the region of 130~160 cM on chromosome 6, the LOD scores obviously decreased from 1970 to 1978, and lowered a little bit from 1978 to 1986. However, the results without outliers do not show the same pattern. There were no LOD scores from chromosomes 3 and 6 higher than 1.0, but the highest LOD scores for chromosomes 9 and 16 were from the analysis of 1978. A common characteristic was that in each one of the four regions the highest LOD score from mean BMI of the three exams was not larger than the maximum highest LOD score obtained from single time point analysis. This implied that using mean BMI of multiple time points might reduce the statistical power.

## Discussion

For complex traits, the conventional LOD threshold for significant linkage may be viewed as too stringent. Suggestive linkage (LOD ≥ 2.2) has been invoked to signify potential linkages, though at a reduced genome-wide significance (statistical evidence that would occur by chance once per genome scan) [9]. In this study LOD ≥ 2.2 was used as a threshold to find QTL candidate regions. Our BMI linkage analysis results confirmed four QTL candidate regions recently reported in literature [5-8]. Furthermore, our studies indicate potential gene × environment (time) and/or gene × gene interactions detected from analyses on longitudinal data.

Although analyzing the mean values of quantitative traits from longitudinal data offers the advantages of simplicity of analytic procedure and overall genetic effect over several years, it may lose power and lose the chance to detect interactions as we show in this study. However, summarizing the overall genetic effect from longitudinal family data is not easy. Several studies [5] have used different methods to perform meta-analyses on various data sources, but these methods are invalid for our study because each measurement is not independent, which violates the assumptions of meta-analysis. Since longitudinal data are very difficult to collect, we suggest reporting results from both mean values and from each measurement until new methods are designed to analyze such data sets.

Detected genetic effects on obesity-related traits are likely to be modified by several factors including 1) actual genetic changes over time (e.g., gene expression can be turned up or down as people age); 2) secular changes over time that are not genetic; and 3) random variation (e.g., measurement error). Our linkage analysis results from BMI in the four chromosomal regions is an example of a mix of the three situations. The consistent suggestion of linkage in three measurements from the Framingham Heart Study and replicated findings from other independent data [5,6,8] and six measurements from the Framingham Heart Study [7] indicate potentially interesting genes on these four chromosomes. The recent BMI linkage analysis of the more complete data in the Framingham Heart Study [7] showed that the peaks of highest LOD scores decreased with age at same location of chromosome 6 (~140 cM) in the first five time points, but in the sixth time point, the location of the highest LOD score was 20 cM away (~166 cM) from previous peaks. Since the data were in the same cohort, the peak at 166 cM may reflect random variation or a type I error rather than a second peak. If so, there is evidence of gene × environment (time) interactions in these more complete data. After removing outliers, there are big influences on chromosomes 3 and 6, and some changes on chromosomes 9 and 16. Allison et al. [10] showed that outliers tend to be very influential in variance component analysis and bias the results. Otherwise, excluding the nonextreme outliers will lose power. For example, after removing the outliers on chromosomes 3 and 6, the peak locations did not change, but the LOD scores dramatically reduced. We need to further consider how to select an appropriate cut-off point for removing extreme outliers.

## Conclusions

In summary, our studies suggest that elaborate analyses of longitudinal data may provide more insight and improve statistical power. The evidence of QTLs on chromosomes 3, 6, 9, and 16 are potentially interesting and the trend of LOD scores on these chromosomes are likely to be due to gene × environment (time) and/or gene × gene interaction.

## Figures and Tables

**Table 1:** Mean, minimum, maximum, sample size and estimated heritability for BMI

**Year**	**Min**	**Max**	**Mean ± SD**	**Heritability (%) ± SE**	**Sample size**
**1970**	13.41	52.11	25.32 ± 4.34	41.19 ± 4.07	2542^A^
	13.41	38.24	25.24 ± 4.00	43.55 ± 4.23	2527
**1978**	15.31	50.49	25.77 ± 4.38	40.55 ± 4.37	2120^A^
	15.31	38.75	25.57 ± 4.05	38.93 ± 4.49	2096
**1986**	16.87	55.68	26.55 ± 4.71	44.80 ± 4.80	1923^A^
	16.87	41.18	26.35 ± 4.30	45.74 ± 4.87	1896
**Mean**	15.84	49.63	25.92 ± 4.30	46.77 ± 3.84	2569^A^
	15.84	37.88	25.74 ± 3.96	41.38 ± 3.72	2545

^A^In each measurement, values were obtained with outliers in the first line and without outliers in the second line.

**Table 2:** Estimated location (cM)/LOD score at the highest peaks in the four chromosomal regions

	**Chromosome**			
	
**Year**	**3**	**6**	**9**	**16**
**1970**	161/0.21	146/3.53	98/0.75	65/0.93^A^
	161/0.00	146/0.43	98/0.52	44/0.79
**1978**	179/1.97	138/1.48	95/2.13	58/2.38^A^
	179/0.19	138/0.92	97/4.32	58/2.22
**1986**	181/2.88	137/1.37	88/2.24	75/2.43^A^
	181/0.93	137/0.23	89/2.35	44/1.18
**Mean**	166/1.45	138/1.61	94/2.14	67/1.17^A^
	166/0.79	140/0.60	96/2.57	44/1.83

^A^In each measurement, values were obtained with outliers in the first line and without outliers in the second line.
